# Is there a consensus for CBCT use in Orthodontics?

**DOI:** 10.1590/2176-9451.19.5.136-149.sar

**Published:** 2014

**Authors:** Daniela G. Garib, Louise Resti Calil, Claudia Resende Leal, Guilherme Janson

**Affiliations:** 1 Associate professor of Orthodontics at the Hospital for Rehabilitation of Craniofacial Anomalies/USP and School of Dentistry - University of São Paulo/Bauru; 2 Masters student in Orthodontics, School of Dentistry - University of São Paulo/Bauru; 3 Masters student in Science of Rehabilitation, Hospital for Rehabilitation of Craniofacial Anomalies/USP; 4 Full professor, Department of Orthodontics, School of Dentistry - University of São Paulo/Bauru

**Keywords:** Orthodontics, Cone-beam computed tomography, Recommendations

## Abstract

This article aims to discuss current evidence and recommendations for cone-beam computed tomography (CBCT) in Orthodontics. In comparison to conventional radiograph, CBCT has higher radiation doses and, for this reason, is not a standard method of diagnosis in Orthodontics. Routine use of CBCT in substitution to conventional radiograph is considered an unaccepted practice. CBCT should be indicated with criteria only after clinical examination has been performed and when the benefits for diagnosis and treatment planning exceed the risks of a greater radiation dose. It should be requested only when there is a potential to provide new information not demonstrated by conventional scans, when it modifies treatment plan or favors treatment execution. The most frequent indication of CBCT in Orthodontics, with some evidence on its clinical efficacy, includes retained/impacted permanent teeth; severe craniofacial anomalies; severe facial discrepancies with indication of orthodontic-surgical treatment; and bone irregularities or malformation of TMJ accompanied by signs and symptoms. In exceptional cases of adult patients when critical tooth movement are planned in regions with deficient buccolingual thickness of the alveolar ridge, CBCT can be indicated provided that there is a perspective of changes in orthodontic treatment planning.

## Abstract (pt)

O presente artigo visa discutir as evidências e recomendações atuais concernentes à indicação da tomografia computadorizada de feixe cônico (TCFC) em Ortodontia. Devido à dose de radiação mais elevada em relação às radiografias, a TCFC não é o método padrão de diagnóstico em Ortodontia. O seu uso rotineiro, em substituição à documentação convencional, é considerado uma prática inaceitável. A TCFC deve ser indicada com muito critério, e somente após uma análise clínica, quando os benefícios para o diagnóstico e tratamento superarem os riscos de uma dose mais elevada de radiação. Deve ser requisitada estritamente quando houver um potencial de prover novas informações não demonstradas em exames radiográficos convencionais, modificando o plano de tratamento ou facilitando a sua execução. As indicações mais frequentes em Ortodontia, que demonstram algum nível de evidência sobre sua eficácia clínica, podem ser resumidas em casos de dentes permanentes retidos; anomalias craniofaciais complexas; discrepâncias faciais severas com indicação de tratamento ortodôntico-cirúrgico; e malformações ou irregularidades ósseas na ATM acompanhadas de sinais e sintomas. Em casos excepcionais, em pacientes adultos em que se planeja movimentos dentários críticos em áreas com espessura óssea vestibulolingual deficiente, a TCFC pode ser indicada, desde que se vislumbre uma perspectiva de alteração no plano de tratamento ortodôntico.

## INTRODUCTION

We have currently been through modern times in Orthodontics. In a retrospective view of our science and art, we envisage a Classical era from the end of the XIX century until the 60s with the legacy of Edward Hartley Angle and his eminent pupils, including Charles Tweed, Broadbent and Brodie.^49^^,^ 50 After the Classical era, a Contemporary era started in the 70s not only with the development of specific occlusal objectives and the Straigth-Wire appliance by Andrews, but also with the development of orthognatic surgery and facial analysis for orthodontic diagnosis.^51^^,^ 52 When we look to the present, we see our time being highlighted by two major vanguard advents: tridimensional images and skeletal anchorage. 

Cone-beam computed tomography (CBCT) together with digital dental models and 3D facial photographs personify the modernity of the present. Introduced in 1998,^39^ CBCT is in its adolescence, but has contributed with over seven hundred international publications in Orthodontics, according to a search at Pubmed database. Evidence related to CBCT have provided important development in three levels: orthodontic diagnosis; orthodontic or orthodontic-surgical treatment planning; and knowledge of treatment outcomes. It is not difficult to fall in love for CBCT scans, once they allow three-dimensional visualization of the morphology of the face and cranium, and demonstrate one's anatomy in multiplanar sections with adequate resolution and sharpness.^21^ CBCT presents high accuracy and precision, sensibility and specificity, as well as absence of image amplification.^6^^,^ 7 ^,^ 9 ^,^ 11 ^,^ 17 ^,^ 27 ^,^ 28 ^,^ 33 ^-^ 36 ^,^ 38 ^,^ 41 ^,^ 43 Faced with these advantages, the following question recurrently arises: Can CBCT be indicated as a routine in Orthodontics?

As every light has its shadows, a method does not have advantages, only. CBCT has the drawback of having a higher radiation dose compared to conventional radiograph frequently requested in Orthodontics.^3^^,^ 45 Effective radiation dose is the sum of the dose received by all irradiated tissues and organs, considering both tissue weight and the quality of ionizing radiation in terms of biological effects.^15^ Effective radiation dose represents a stochastic risk to health, in other words, the probability of carcinogenesis and genetic effects on irradiated tissues.^15^ During X-ray examination, millions of photons pass through patient's cells and can cause damage to DNA molecules due to ionization.^15^ The majority of changes caused to genetic material is reversible and immediately repaired.^15^ However, DNA may be rarely, yet permanently altered, thereby establishing a genetic mutation.^15^ Fortunately, effective dose and risks related to dental radiation are very small compared to the natural risks of carcinogenesis.^15^^,^ 16 Nevertheless, some limited evidence on the increase of radiation-related tumor in the brain and thyroid glands requires caution and rationality before indicating X-ray examination in Dentistry, including conventional radiographs.^15^ This concern is amplified in children, as they present tissues with higher radiosensitivity, greater number of cell divisions and a longer lifetime spam for carcinogenesis development.^16^

The effective radiation dose of CBCT depends on the scanner, the field of view (FOV) and on the acquisition protocol, particularly considering resolution or voxel dimension.^3^ For a detailed analysis of CBCT effective dose, we recommend consulting Table 5 of the manuscript issued by the American Academy of Oral and Maxilofacial Radiology, published in 2013 with the goal of discussing CBCT recommendations in Orthodontics.^3^ The aforementioned table also compares the effective radiation dose of extraoral radiographs and multi-slice computed tomography. These data are summarized in Table 1.

**Table 1:** Effective radiation dose (EICRP 2007) expressed in microSieverts (mSv) and produced by cone-beam computed tomography at different resolutions and fields of view (FOV) in comparison with multi-slice CT and conventional radiograph. Data adapted from the American Academy of Oral and Maxillofacial Radiology (2013).3 Great variation in radiation dose according to each type of scan occurs due to differences caused by the scanner and the acquisition protocol.

EXAMINATION	Effective dose (mSv)
CBCT of face and cranium (FOV > 15 cm)	52 to 1073
CBCT of face (FOV 10 - 15 cm)	61 to 603
CBCT of the jaws (FOV < 10 cm)	18 to 333
Multi-slice CT	426 to 1160
Panoramic radiograph	6 to 50
Cephalogram	2 to 10

**Table 2:** Basic principles to be followed in daily clinical practice before requesting cone-beam computed tomography

Principle 1	CBCT should not be used routinely for all patients.
Principle 2	CBCT examinations must not be carried out unless a history and clinical examination have been performed.
Principle 3	CBCT examinations must be justified for each patient.
Principle 4	CBCT field of view (FOV) should be restricted as much as possible.
Principle 5	The lowest achievable resolution should be used without jeopardizing evaluation of the area of interest.

**Table 3:** CBCT recommendations for orthodontic purposes, according to the American Academy of Oral and Maxillofacial Radiology (AAOMR).3

Localization of impacted teeth and identification of associated root resorption* » CBCT should only be used when Multi-slice CT is necessary, in which case CBCT is preferred due to lower radiation dose; or » CBCT should only be used when the question for which imaging is required cannot be answered adequately by lower dose conventional (traditional) radiograph;
Clef lip/palate* » CBCT should only be used when Helicoidal CT is necessary, in which case CBCT is preferred due to lower radiation dose;
Mini-implants: Proper mini-implant placement site* » CBCT are rarely necessary, except for cases with critical space left for mini-implant placement;
**Severe cases of skeletal discrepancies** » CBCT of the face might be used to develop orthosurgical treatment planning; » Preference is given to patients older than 16 years of age;
**Pre-surgical assessment of impacted teeth** » CBCT should only be used when the question for which imaging is required cannot be answered adequately by lower dose conventional (traditional) radiography;
**Orthognathic surgery planning** » CBCT of the face might be used to develop orthosurgical treatment planning;
**TMJ assessment** » CBCT should only be used when Helicoidal CT is necessary, in which case CBCT is preferred due to lower radiation dose;

**Table 4:** CBCT recommendations in Orthodontics according to the European SedentexCT (2012) guidelines.15 *field of view should be as restricted as possible.

Localization of impacted teeth and identification of associated root resorption* » CBCT should only be used when Multi-slice CT is necessary, in which case CBCT is preferred due to lower radiation dose; or » CBCT should only be used when the question for which imaging is required cannot be answered adequately by lower dose conventional (traditional) radiograph;
Clef lip/palate* » CBCT should only be used when Helicoidal CT is necessary, in which case CBCT is preferred due to lower radiation dose;
Mini-implants: Proper mini-implant placement site* » CBCT are rarely necessary, except for cases with critical space left for mini-implant placement;
**Severe cases of skeletal discrepancies** » CBCT of the face might be used to develop orthosurgical treatment planning; » Preference is given to patients older than 16 years of age;
**Pre-surgical assessment of impacted teeth** » CBCT should only be used when the question for which imaging is required cannot be answered adequately by lower dose conventional (traditional) radiography;
**Orthognathic surgery planning** » CBCT of the face might be used to develop orthosurgical treatment planning;
**TMJ assessment** » CBCT should only be used when Helicoidal CT is necessary, in which case CBCT is preferred due to lower radiation dose;

**Table 5:** Cone-beam computed tomography might be indicated in the aforementioned orthodontic cases, whenever potential benefits of diagnosis, treatment planning and treatment execution outweigh potential risks.

**Eruptive disorders: impacted teeth**
**Severe craniofacial anomalies**
**Severe facial discrepancies** potentially subjected to orthosurgical treatment
**Bone irregularities or malformation of TMJ**
**Deficient buccolingual thickness of the alveolar ridge ** In exceptional cases of adult patients potentially subject to critical tooth movement in areas of deficient bone, CBCT is indicated provided that there is a perspective of changes in treatment planning

By weighing the advantages and risks of CBCT and based on specialized and updated literature, this article aims to discuss CBCT use in Orthodontics. The main goal of this paper is to guide the orthodontist towards a discerning use of CBCT in daily practice.

## THE CONTROVERSY

In November, 2010, a publication in "The New York Times" reported the abuse of dental professionals in indicating CBCT to children and adolescents.^8^ The article had great impact in the United States and encouraged the American Association of Orthodontics and the American Academy of Oral and Maxillofacial Radiology to prepare guidelines for CBCT use in Orthodontics.^3^ During the 3-year interval between these two publications, much controversy was seen on this subject. 

In 2011, 83% of postgraduate programs in Orthodontics in the US and Canada reported to use CBCT.^46^ The majority (82%) of them recommended CBCT only in selected cases, including impacted teeth (100% of programs), craniofacial anomalies (100% of programs) and TMJ (67%) or upper airway assessment (28%). Only 18% of programs reported replacing conventional radiograph by CBCT. Most of them, however, routinely used conventional radiograph for control during orthodontic treatment.

CBCT recommendation in Orthodontics raised so much controversy that the American Journal of Orthodontics and Dentofacial Orthopedics published a Point-Counterpoint session on the subject in 2012*.* 23 ^,^ 29 On one side, in defense of routine use of CBCT for comprehensive orthodontic treatment, was Dr. Brent Larson, director of the Orthodontic division of the University of Minnesota, United States.^29^ On the other side, against the idea of routine use of CBCT for comprehensive orthodontic treatment, was Dr. Demetrius Halazonetis from the University of Athens, Greece.^23^ The aforementioned publication also portraits the dichotomy between United States and Europe concerning the conservative approach of CBCT use.

Defense was based on arguments such as increased geometrical accuracy and reliability of measurements on CBCT images; high sensitivity for localization of impacted teeth and identification of related root resorption; easiness in quantifying discrepancies in cases of facial asymmetry; sharp visualization of TMJ, upper airway and tooth buccal and lingual bone plates; significant frequency (10%) of incidental findings; ease in mini-implant and customized fixed appliance planning; confidence provided by CBCT to therapeutic choices; the possibility to simulate and demonstrate the therapy of choice to patients; and last but not least, the evidence that CBCT radiation dose is minimal in comparison to the sum of radiation doses of panoramic radiograph, cephalometric radiograph and the full set of periapical radiographs.^29^

Opposing to the general use of CBCT in Orthodontics, it was mentioned that criteria for patients selection should be based on the ratio risk-benefit of CBCT; and that there was not enough evidence supporting CBCT efficacy for diagnosis, treatment planning or treatment outcomes in Corrective Orthodontics.^23^ We invite readers to advance in the arguments raised by Dr. Halazonetis^23^ by carefully examining the following topics of this article.

## WEIGHING RISKS AND BENEFITS

**Figure 1:**
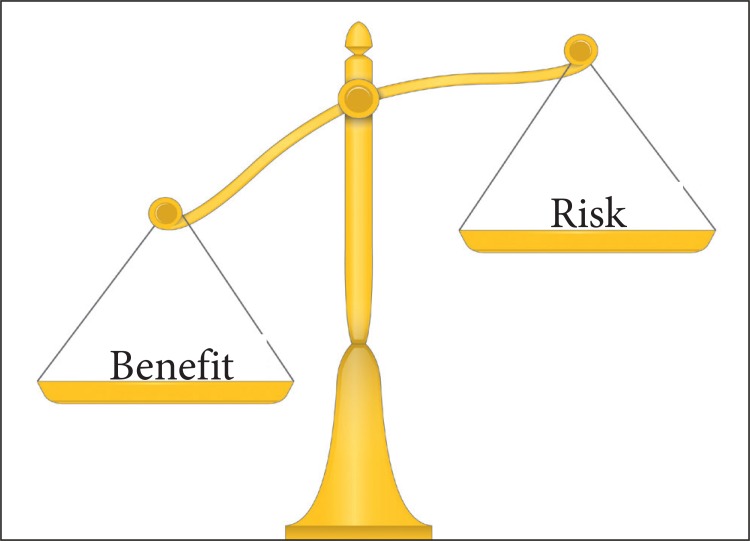
Cone-beam computed tomography should only be requested in cases in which the potential benefits of diagnosis and treatment planning, treatment execution or treatment outcomes outweigh the potential risks of an increased radiation dose

There seems to be an antithesis between what the orthodontist desires and what the orthodontist can do with regard to CBCT. The conflict starts in clinicians' attraction to visualize the virtual anatomical replica of the patient at high resolution; however, the risk related to increased radiation dose is rationalized. The Golden Law of Ethics says that we should do to others only what we would like to do to ourselves. Therefore, before requesting a CBCT scan, the orthodontist should weigh the risks and benefits. CBCT scans should only be requested in cases in which the potential benefits of diagnosis and treatment planning, treatment execution or treatment outcomes outweigh the potential risks of an increased radiation dose (Fig 1).

The benefit for orthodontic diagnosis can be analyzed by the capacity of CBCT scans to change orthodontic treatment planning. Another benefit of CBCT would be to favor treatment execution, as observed in cases in need of orthognatic surgery or implants in which the surgeon performs a 3D simulation with the goal of performing the surgery *in vivo *with more precision. Finally, a long-term benefit would be to have better or more efficient treatment outcomes compared to treatment outcomes reached without CBCT images. Evidence in these three levels of benefits guide the recommendations for CBCT use in Dentistry, as recently published by committees in North America and Europe^3^^,^ 15 and which we are about to discuss in the next topic of this article.

## BASIC PRINCIPLES FOR CBCT RECOMMENDATION

According to the American Academy of Oral and Maxillofacial Radiology, there is neither convincing evidence for radiation-induced carcinogenesis at the level of dental exposure, nor absence of evidence of such effect. Because Orthodontics is a field of health, we prudently assume there is a risk, given that there is no safe limit for ionizing radiation.^3^ Each exposure has a cumulative effect on the risk of carcinogenesis.^3^ In this perspective, the basic principles recommended by European and North-American guidelines aim to avoid or minimize unnecessary exposure for diagnosis purposes. 

**Figure 2:**
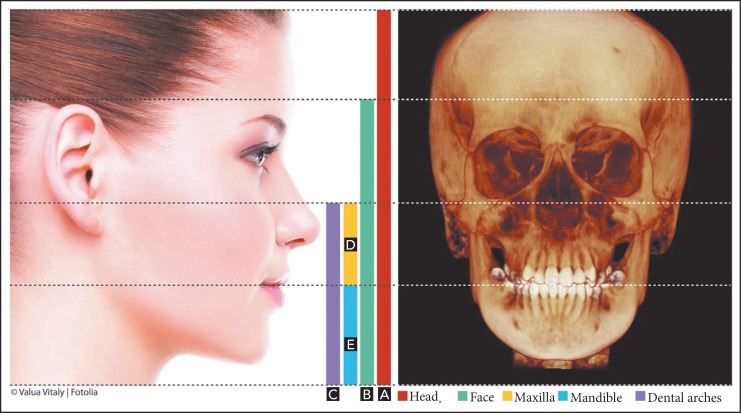
Cone-beam computed tomography field of view: It is cylindrical and determined according to its vertical extent. A) Field of view of the face and cranium; B) Field of view of the face; C) Field of view of the jaws: D) Field of view of the maxilla; E) Field of view of the mandible.

**Figure 3:**
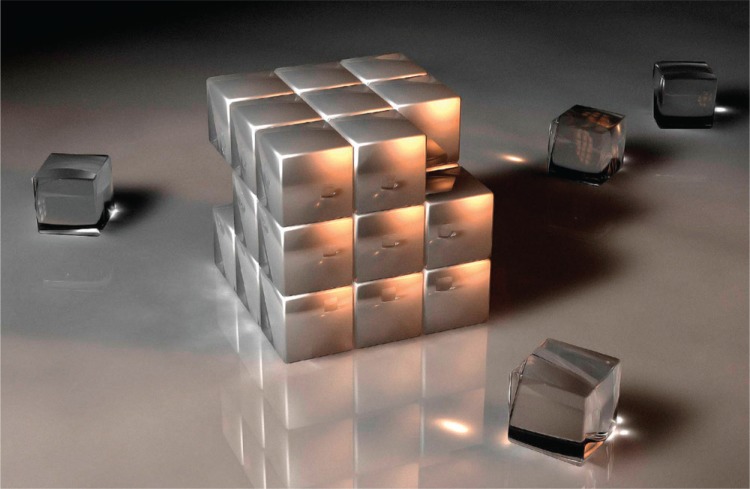
The voxel is cubic-shaped and is the smallest unit of a tomographic image. In CBCT, voxels have equal and submillimetric dimensions in height, width and depth.

The orthodontist should follow some basic principles regarding indication of cone-beam computed tomography, as described bellow and summarized in Table 2:

1. Indiscriminate, routine use of CBCT for all orthodontic patients is considered an unacceptable practice.^15^

2. CBCT examination must not be carried out unless a history and clinical examination have been performed.^3^^,^ 15

3. CBCT examinations must be justified for each patient. CBCT scans should only be requested when there is a potential for CBCT images to provide new information not provided by conventional radiograph.^15^ Clinical justification should be based on the risk-benefit ratio of radiation exposure.^44^ This principle opens up space for discussion and controversy, once the benefits of CBCT are not clear for all possible orthodontic indications. There is lack of evidence on the benefits for diagnosis, treatment planning, treatment execution or treatment outcomes in the orthodontic literature.

4. CBCT field of view (FOV) should be restricted as much as possible.^15^ The field of view is the vertical volume covered by the exam. It is cylindrical, varies in height and can be adjusted before the exam. Thus, CBCT can be requested with a small (maxilla or mandible), medium (maxilla and mandible) or large (face and cranium) field of view, as illustrated in Figure 2. The greater the field of view, the greater the radiation dose. Therefore, the exam should include only the areas of interest for diagnosis so as to minimize radiation dose and follow the ALARA principle (As Low As Reasonably Achievable*).*

5. To use the lowest achievable resolution possible without jeopardizing evaluation of the area of interest.^3^^,^ 15 CBCT image resolution is influenced, among other factors, by voxel dimension. The voxel is the smallest unit of a tomographic image. The word "voxel" is the combination of the words "volume" and "pixel". Voxels are cubic-shaped and have equal and submillimetric dimensions in height, width and depth (Fig 3). Voxel size may vary from 0.1 to 0.4 mm, and the smaller the voxel dimension, the better the spatial resolution, but the greater the radiation dose.^34^ CBCT scans with high resolution (0.1 mm or 0.2 mm voxel size) should only be requested when in need of visualization of small details and delicate structures, such as mild root resorption, bone dehiscence and tooth fracture. When the purpose of the exam does not involve a high level of detail, voxel sizes of 0.3 mm and 0.4 mm should be preferred. 

## CLINICAL RECOMMENDATION IN ORTHODONTICS

**Figure 4:**
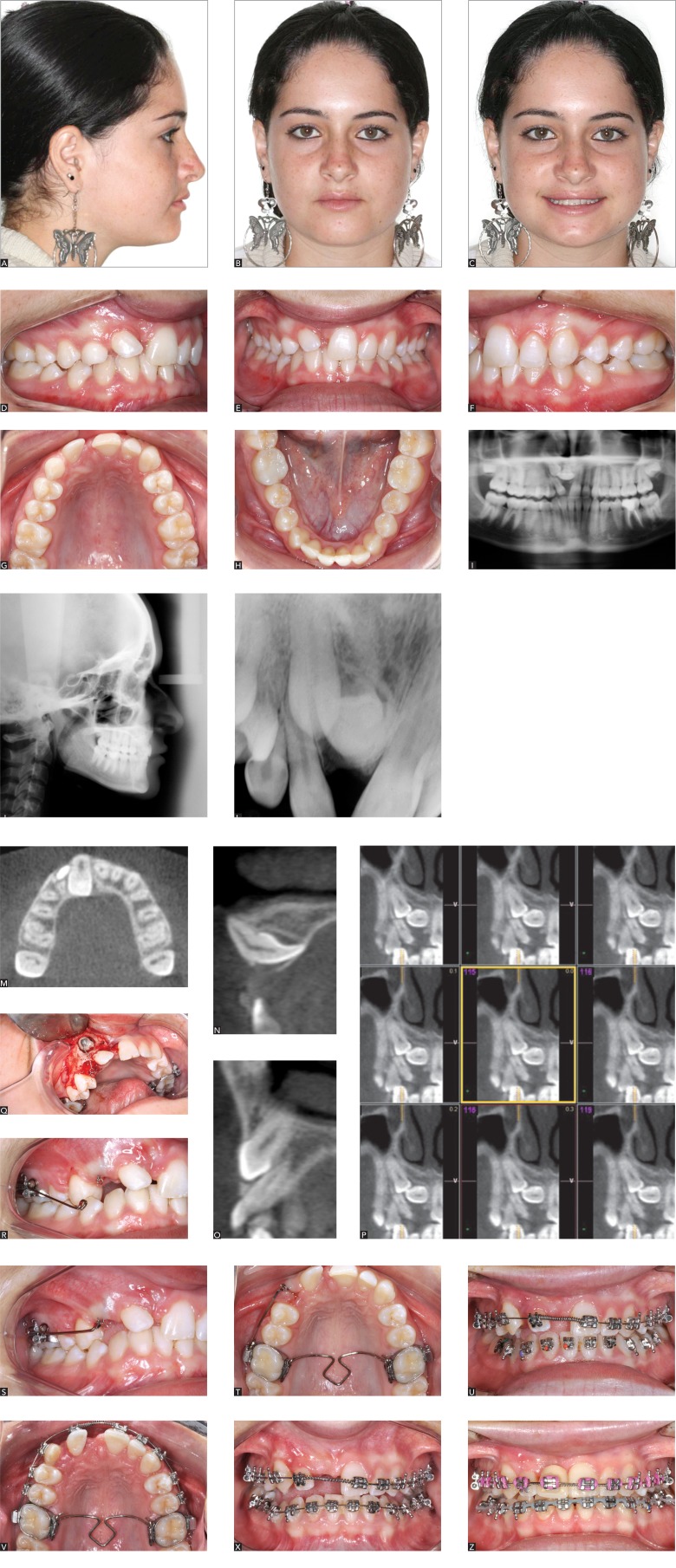
A-H) Severe case of a 15-year-old patient with central incisor and maxillary canine retention on the left side (# 11 and 13). I, J, L) Conventional radiograph included in patient's orthodontic records confirms #11 and 13 retention. M) Axial CBCT scan revealing proximity between retained teeth and the tipped lateral incisor root. N, O) Cross-sectional slices revealing canine buccaly positioned, as well as central incisor atypically positioned with the incisal surface posteriorly faced, and the presence of root dilaceration. P, Q) Treatment began by tractioning #13 by means of occlusal and buccal force applied to preserve #12 root. R, S) Regaining space in the region of right central incisor for further traction carried out by applying occlusal and buccal force aimed at repositioning the incisal edge at the center of the alveolar ridge; T a Z) After an unsuccessful attempt to traction #11, extraction and anterior rehabilitation were recommended. (Treatment performed by Dr. Marilia Yatabe and Dr. Marcos Ioshida, postgraduate students at FOB-USP).

Based on principles 1 and 3 of the previous topic, the orthodontist should critically assess the risk-benefit ratio of CBCT exam before requesting it. In general, the decision regarding the use of CBCT depends on the severity of malocclusion^3^. The more severe the malocclusion, the more probability of needing the examination (Fig 4). On the other hand, the milder the malocclusion, the less likelihood of needing a CBCT scan. Malocclusion severity is understood as the presence of vertical and sagittal skeletal discrepancies, facial asymmetry, craniofacial malformation and tooth eruptive disorders. There is no rationale in indicating CBCT for patients with Class I malocclusion and anterior crowding, for example. In these cases, CT scans would not have the potential to change diagnosis, prognosis and treatment planning. In contrast, a patient with severe skeletal discrepancy or craniofacial anomalies in need of surgical-orthodontic treatment could have a more accurate diagnosis and prognosis, a more specific treatment planning as well as easy treatment execution with a qualitative increase in treatment outcomes. Additionally, the decision on requiring a CBCT scan is age-dependent.^3^ The younger the patient, the more critical should the professional be for indicating a CBCT exam, particularly due to the biological effects of exposure to radiation.^3^

CBCT recommendation in Dentistry is based on a general evaluation of the benefits in counterpoint to risks.^44^ However, how can the benefits of CBCT be evaluated? Benefits can be understood as the method efficacy. Imaging examinations present six levels of efficacy:^15^^,^ 23 technical efficacy related to the quality of images; diagnosis efficacy understood as the low frequency of false-negative and false-positive diagnosis or accuracy and reproducibility of quantitative analyses; diagnostic thinking efficacy related to the capacity of the method to change a pre-established diagnosis; therapeutic accuracy representing the potential of the exam to change treatment planning; orthodontic finishing efficacy taking into account the qualitative gain of treatment results; and, finally, the societal efficacy.^23^

In Orthodontics, there is few evidence on the CBCT potential to change the quality of treatment outcomes and no evidence of CBCT social benefits.^23^ Current evidence of efficacy for the other four levels have guided the North-American and European recommendations for CBCT use. In other words, evidence of efficacy guided the eligibility criteria of cases that justify the use of CBCT.

The North-American guidelines for CBCT use in Orthodontics were published in 2013 with the coordination of the American Academy of Oral and Maxillofacial Radiology (AAOMR) and have remained in force for 5 years.^3^ Table 3 shows the orthodontic indications of CBCT according to the American guidelines.^3^

The European evidenced-based guidelines, known as SedentexCT Project, were issued in 2012^15^ and were more conservative regarding the use of CBCT in Orthodontics. Table 4 summarizes the conclusion of these guidelines with regard to orthodontic cases. The difference between North-American and European recommendations may be explained by the distinct criteria used. The North-American guidelines were based on the most frequent use of CBCT revealed in the literature. Conversely, the SedentexCT guidelines were strictly based on the presence of high levels of evidence on CBCT efficacy.

## DISCUSSING AND DRAWING CONCLUSIONS TOWARDS CLINICAL RECOMMENDATIONS

In the diagnosis of impacted teeth, CT scans are advantageous for providing the exact tridimensional location of the crown and the root(s) of unerupted teeth and their relationship with neighboring teeth. CBCT scans might also reveal the presence of associated root resorption in neighboring teeth, even when resorption lacunae are bucally or lingually located.^1^ CT scans are more sensitive in comparison to conventional radiograph when diagnosing resorption of impacted teeth.^1^ Conventional radiograph, including the periapical one, is limited in terms of overlapping of bucally or lingually impacted teeth images and neighboring teeth roots. For this reason, periapical radiograph might lead to false-negative results, even in the presence of deep root resorption reaching the root canal.^14^

Identifying root resorption in teeth neighboring impacted canines might alter treatment planning in a significant number of cases.^24^^,^ 26 There is evidence highlighting that CT scans might alter treatment planning in approximately 30% of cases.^24^ For instance, in a case with previously planned extraction of maxillary premolars, identifying the presence of root resorption in lateral incisors might lead to extraction of anterior teeth instead of posterior. Furthermore, CT scans might lead to better planning of traction force direction.^24^ Surgical exposure and bonding for traction of impacted teeth might also benefit from accurate positional diagnosis provided by CT scans.^24^

The aforementioned benefits yielded by CBCT for impacted teeth allow orthodontists to be more confident in diagnosing and performing treatment plan.^24^ Lastly, it has been recently proved that CBCT renders treatment of complexly positioned impacted canines easier, thereby reducing treatment time.^2^

Cases in which diagnosis of impacted teeth is made in initial conventional orthodontic records, CBCT might be requested as a compliment. Should that be the case, CBCT scan protocols should include a partial field of view comprising the maxilla or the mandible, only.^15^ A reduced field of view minimizes exposure to radiation. Doubts involving cases of impacted teeth are usually solved by serial axial and cross-sectional slices of volumetric 3D reconstruction. Importantly, axial slices are the most appropriate CBCT scans used for diagnosis of root resorption associated with impacted teeth. Cross-sectional slices sometimes fail to show the entire cervico-apical portion of the roots, especially due to mesiodistal tooth angulation. Additionally, they might give a false impression of inexistent root resorption. 

**Figure 5:**
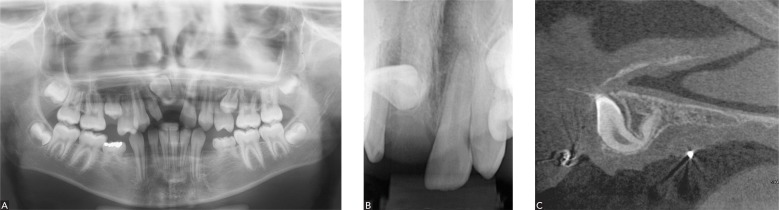
Retention of right maxillary central incisor caused by trauma during childhood. A, B) Conventional radiograph. C) CT scans revealed dilaceration associated with root suffocation, both of which were not identified by conventional radiograph. The unfavorable root condition enlightened treatment prognosis and influenced orthodontic planning.

The literature does not highlight studies validating CBCT as a diagnosis tool of ankylosis of impacted teeth, perhaps due to difficulties in finding methods to investigate the theme. Cases in which the periodontal ligament cannot be identified by CBCT slices do not necessarily involve ankylosis. The periodontal ligament is on average 0.2-mm thick. For this reason, high resolution scans are required for its identification. Unlike ankylosis, root fracture is easily diagnosed by CBCT scans.^15^ Cases of permanent impacted teeth are benefited from CBCT when conventional radiograph does not provide enough information for diagnosis, prognosis, treatment plan, surgical intervention and orthodontic therapy (Fig 5). 

As for CBCT use in cases of cleft lip/palate, although some studies have assessed alveolar bone graft outcomes by means of computed tomography,^40^ there is no evidence proving that this assessment method influences orthosurgical treatment protocol in daily practice. Empirically, the benefits of CBCT use are acknowledged for diagnosis and surgical treatment of more severe craniofacial anomalies with malformation of the midface, mandible or TMJ, particularly involving facial asymmetry. In these cases, CBCT is beneficial for allowing identification of the exact location of morphological errors, three-dimensionally quantifying the error and providing therapeutic planning that includes osteogenic distraction or craniofacial surgery. 

Cone-beam computed tomography is indicated for orthodontic cases that require analysis of TMJ bone components accompanied by signs and symptoms.^3^^,^ 44 CBCT programs reconstruct TMJ sequential slices, both in latero-lateral and anterior-posterior axes, and provide clear imaging of articular fossa and condyles. Morphological analysis of CT scans might reveal the presence of erosions, ankylosis, hyperplasia/hypoplasia of the condyle or degenerative arthritis.^5^^,^ 25 In comparison to panoramic radiograph and linear tomography, however, CBCT proves more accurate in diagnosing erosion of the condyle.^25^ Conventional radiograph is quite limited in reproducing TMJ morphology due to imaging overlap.^25^ Nevertheless, TMJ imaging is not necessary for the diagnosis of temporomandibular disorders.^42^ Furthermore, CBCT proves a good method to assess TMJ after orthognathic surgery, particularly when there is considerable potential for resorption of the condyle.^10^ Based on such evidence, CBCT use is appropriate for diagnosis and development of treatment planning of TMJ skeletal irregularities accompanied by signs and symptoms.

Orthognathic surgery and its outcomes might benefit from CBCT scans at the time of diagnosis.^12^ Additionally, CBCT is recommended in cases of severe facial skeletal discrepancies that require orthosurgical treatment.^3^^,^ 15

However, would CBCT be useful to assess one's airway? CBCT proves advantageous to assess upper airways in terms of sagittal and transverse linear measurements as well as calculation of airway total area and volume.^30^ However, the method has its limitations. CBCT airway imaging might vary according to patient's swallowing movement and position during the exam.^30^ Whenever the patient swallows, the soft palate is lifted, which causes the nasopharynx to distort. Furthermore, some CBCT scanners require the patient to be in supine position, while others require the patient to remain sited or standing. Different scanners register different images of upper airways due to soft palate mobility.^13^ Moreover, static analysis of patient's airways is another limitation posed by CBCT which differs from videofluoroscopy, as the latter allows a dynamic pharyngeal analysis. Additionally, the ideal method used to diagnose obstructive sleep apnea syndrome is polysomnography instead of CBCT. Previous studies found significant correlation between profile cephalogram and CBCT used to analyze patient's airways area and volume.^48^ Nasopharyngeal sagittal linear measurement is strongly correlated to volume of upper airways.^30^ Thus, despite building a 2D representation of a 3D structure such as patient's airways, profile cephalogram remains as a reliable method used to assess pharyngeal obstruction. To date, there seems to be no evidence stating that CBCT 3D imaging of one's airways affects orthodontic diagnosis and treatment. Therefore, there is no point in requesting CBCT scans with a view to tridimensionally assessing upper airways for orthodontic purposes. 

Finally, it seems to be important to discuss the indication of CT scans to assess alveolar bone limits for tooth movement. One of the advantages of computed tomography used for orthodontic purposes is related to its ability of providing images of the alveolar bone which bucally and lingually surrounds the teeth. The only imaging diagnosis methods available to assess and measure buccal and lingual bone plates are multi-slice computed tomography and cone-beam computed tomography. Before computed tomography, patient's buccal and lingual bone plates could not be assessed by conventional radiograph due to imaging overlap and gingival covering. In the 90s, multi-slice computed tomography was validated to assess buccal and lingual alveolar bone.^20^ Bone plates thinner than 0.2 mm were not always shown by multi-slice TC scans.^20^ Additionally, cadaver studies revealed that buccal and lingual horizontal bone defects were assessed by multi-slice TC scans, but could not be identified by periapical radiograph.^19^ Moreover, an experimental study in which bone dehiscence was artificially caused in cadaver jaws concluded that CT scans were the only imaging diagnosis method capable of quantitatively assessing alveolar ridge as well as buccal/lingual bone plates buccolingual thickness.^18^

After cone-beam computed tomography was introduced,^39^ new studies were conducted to validate the method with a view to assessing buccolingual alveolar bone. Misch, Yi and Sarment^35^ measured buccal bone defects and found a mean difference of 0.4 mm (SD = 1.2) between direct measurements performed on dry skulls and CBCT scans taken by an iCAT scanner. Mol and Balasundaram^36^ evaluated accuracy of buccal/lingual bone plate measurements performed in cross-sectional CBCT slices acquired by NewTom QR-DVT-9000. They found a mean difference of -0.23 mm between real measurements and CBCT, thereby revealing that CBCT tends to underestimate real bone loss. The mean absolute difference between anatomic measurements and CBCT scans was 1.27 mm (SD = 1.43). Lower incisors had the lowest accuracy. The magnitude of the error was attributed to the use of primitive CBCT scanners which are no longer available. The devices produced unclear, low-contrast images.

Lund, Gröndahl and Gröndahl^33^ used cross-sectional CBCT slices of a dry scull scanned by Accuitomo scanner (Morita, Kyoto, Japan) to measure buccal/lingual bone plates. The mean error for the distance between the cementoenamel junction and the bone crest was -0.04 mm (SD = 0.54), with variation between -1.5 mm and +1.9 mm.

Leung et al^31^ assessed accuracy of natural bone dehiscence measurements and CBCT sensitivity of identifying them. The authors used 13 dry skull scans acquired by CB MercuRay (Hitachi, Medical Systems American, Ohio, USA). Their study presented some negative morphological aspects, as bone dehiscence was assessed in 3D reconstruction instead of CBCT orthogonal slices. Furthermore, they measured the distance from cuspid tips to the alveolar bone crest instead of the distance between the cementoenamel junction and the bone crest. The authors found a mean difference of -0.2 mm (SD = 1.0) and an absolute difference of 0.6 mm (SD = 0.8 mm) between real and digital measurements. They concluded that 3D reconstructions present low sensitivity (0.4), but high specificity (0.95) in identifying bone dehiscence. 

Despite submillimetric accuracy revealed by CBCT, some principles must be followed when assessing buccal/lingual bone plates.^37^ Imaging spatial resolution is the minimal distance required to distinguish two contiguous anatomical structures.^37 ^The smaller the anatomical structures, the higher the spatial resolution required.^37^ Spatial resolution is not equivalent to voxel size (the smallest tomographic image), since calculation of mean partial volume, noise and artifacts negatively influence imaging clearness.^37^ Mean partial volume occurs when a voxel includes two structures of different densities, for instance, the periodontal ligament and the alveolar bone. Density attributed to the voxel will be equivalent to the mean density of both tissues,^44^ which hinders clear visualization of the limits of each structure in computed tomography. 

Images acquired by iCAT scanner with voxel size of 0.2 mm have a mean spatial resolution of 0.4 mm, whereas images with voxel size of 0.3 and 0.4 mm have a spatial resolution of 0.7 mm.^4^ Bone plates thinner than the imaging spatial resolution might not be revealed by CBCT, thereby reaching a false-positive diagnosis of bone dehiscence or achieving quantitative assessments that underestimate the level of bone crest.^47^ Thus, care should be taken while drawing conclusions based on dimensions smaller than the imaging spatial resolution.^37^ In Orthodontics, voxel sizes of 0.4 mm and 0.3 mm are the most used.^47^ However, investigations aiming to assess periodontal structures before and/or after orthodontic treatment should use the smallest voxel possible.^37^ The smallest voxel in iCAT scanner is 0.2 mm; whereas Accuitomo and PreXon scanners produce images with higher spatial resolution, as their smallest voxel is 0.1 mm^32^ Images with reduced voxel size are more accurate in terms of thickness and height of buccal/lingual bone plates.^47^

Therefore, CBCT scans are useful to assess the presence of bone dehiscence. However, CBCT scans have been restricted to investigations that guide the clinician towards the alveolar limits in cases of critical movement such as buccolingual tooth movement.^22^ In Orthodontics, CBCT should be indicated to assess deficiencies of buccolingual thickness in the alveolar ridge of adult patients subjected to critical tooth movement in which case absence of buccolingual bone would affect orthodontic treatment. In these cases, the best option would be to use high resolution (reduced voxels) and a limited field of view (FOV) (Table 5).

## IMPORTANT RECOMMENDATIONS: EDUCATION AND TRAINING

According to SedentexCT guidelines,^15^ the prescriber, the clinics where the exam is taken and the medical physics expert share the responsibility over a radiographic exam. All professionals involved with CBCT, including the prescriber, should receive theoretical and practical training that includes the technical procedure of image acquisition, radiation dose, radiation protection and tomographic reading.^15^ That is, the prescriber should know when and for what purpose he will request it. Furthermore, he should know how to exam and fully interpret it.

## FINAL CONSIDERATIONS

Cone-beam computed tomography is not a standard diagnosis method in Orthodontics. CBCT should be indicated with criteria, when the potential benefits for diagnosis and treatment planning outweigh the potential risks of an increased radiation dose. The recommendations discussed in this article originate from current evidence and therefore are time-dependent. In the future, new evidence as well as technological evolution and innovation of CBCT scanners could change the current indications of CBCT in Orthodontics. 
